# Niagara and Orleans County Veterinary Medical Society

**Published:** 1897-02

**Authors:** 


### NIAGARA AND ORLEANS COUNTY VETERINARY MEDICAL SOCIETY.

A MEETING of this Society was held at Niagara, January 9th. Members present: Drs. Martin and Crowforth, of Lockport; Williams, of Middleport; Moore, of Wilson; and Thomson, of Niagara. Matters pertaining to the organization were the principal subjects of the deliberations. Next meeting at Medina in April.

